# Distinct Gut Microbiota and Metabolite Profiles Induced by Different Feeding Methods in Healthy Chinese Infants

**DOI:** 10.3389/fmicb.2020.00714

**Published:** 2020-05-06

**Authors:** Na Li, Fenfen Yan, Nana Wang, Yue Song, Yingxue Yue, Jiaqi Guan, Bailiang Li, Guicheng Huo

**Affiliations:** ^1^Key Laboratory of Dairy Science, Ministry of Education, Northeast Agricultural University, Harbin, China; ^2^College of Food, Northeast Agricultural University, Harbin, China

**Keywords:** infant, human milk, formula milk, complementary food, microbiota, metabolite

## Abstract

Human milk is closely correlated with infant gut microbiota and is important for infant development. However, most infants receive exclusively insufficient breast milk, and the discordance between effects of commercial formula and human milk exists. To elucidate the differences induced by various feeding methods, we determined microbiota and metabolites composition in fecal samples from 77 healthy infants in Northeast China and identified the differences in various feeding methods. Bacterial 16S rRNA gene sequence analysis demonstrated that the fecal samples of exclusively breastfed (BF) infants were abundant in *Bifidobacterium* and *Lactobacillus*; the mixed-fed (MF) infants had the highest abundance of *Veillonella* and *Klebsiella*; the exclusively formula-fed (FF) infants were enriched in *Bacteroides* and *Blautia*; and the complementary food-fed (CF) infants were associated with higher relative abundance of *Lachnoclostridium* and *Akkermansia*. Liquid chromatography–mass spectrometry (LC-MS)-based metabolomics data revealed that the fecal samples of BF infants had the highest abundance of dl-citrulline, threonine, l-proline, l-glutamine, guanine, and l-arginine; the MF infants were abundant in d-maltose, stearidonic acid, capric acid, and myristic acid; the FF infants were enriched in itaconic acid, 4-pyridoxic acid, prostaglandin B2, thymine, dl-α-hydroxybutyric acid, and orotic acid; and the CF infants were associated with higher relative abundance of taurine, l-tyrosine, adenine, and uric acid. Furthermore, compared with the BF infants, the MF and FF infants were more abundant in fatty acid biosynthesis. Collectively, these findings will provide probable explanations for some of the risks and benefits related to infant feeding methods and will support a theoretical basis for the development of infant formula.

**Graphical Abstract:**
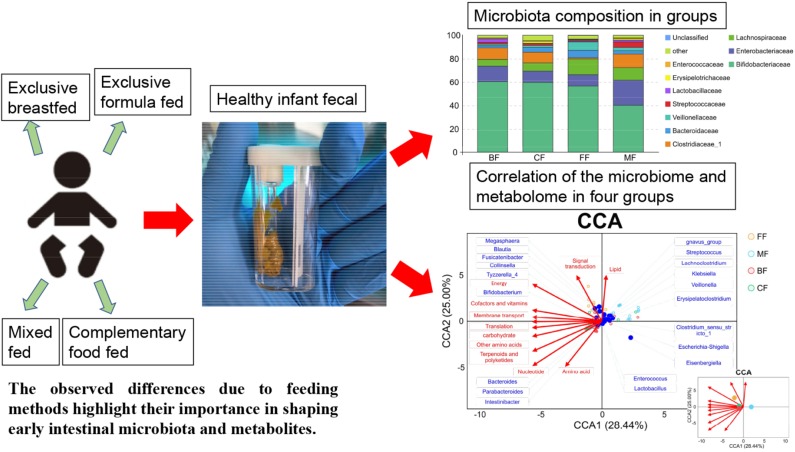
Distinct gut microbiota and metabolite profiles induced by different feeding methods.

## Introduction

The establishment of stable gut microbiota communities closely parallels host growth and immune development in early life (Harald et al., 2012) and has substantial implications for subsequent health (Rautava, 2016). The development of gut microbiota constitutes a highly dynamic and non-random process, in which positive and negative interactions between key microbial taxa take place (Avershina et al., 2013, 2016). This process is influenced by various conditions, such as mode of delivery (Biasucci et al., 2010; Dominguez-Bello et al., 2010; Munyaka et al., 2014), infant feeding method (Bezirtzoglou et al., 2011; Tanya et al., 2012; O'sullivan et al., 2015), gestational ages at birth (Ruiz et al., 2016), maternal diet (Lundgren et al., 2018), environment (Marie-Claire et al., 2014; Sabesfiguera et al., 2015), and host genetics (Benson et al., 2010; Leamy et al., 2014; Turpin et al., 2016). Feeding method is one of the most influential factors that determine early gut microbiota composition (Tanya et al., 2012; O'sullivan et al., 2015) and metabolites (Isabelle et al., 2010).

Breast milk feeding has been associated with strong protection against diarrhea morbidity and mortality (Lamberti et al., 2011) and decreases asthma (Klopp et al., 2017), inflammatory bowel disease (Xu et al., 2017), diabetes (Cardwell et al., 2012; Horta et al., 2015), and obesity (Forbes et al., 2018) as compared with those in non-breast milk-fed (non-BF) or formula-fed (FF) infants, possibly partly because of its effects on the infant gut microbiota. Although formulas closely approximate breast milk composition (Zhang et al., 2013; Schwarzenberg and Georgieff, 2018) and galacto-oligosaccharides and fructo-oligosaccharides (GOS and FOS, respectively) are designed to mimic human milk oligosaccharides (Oozeer et al., 2013), BF and FF infant gut microbiomes remain distinct (Bäckhed et al., 2015; Schwarzenberg and Georgieff, 2018). Formula feeding may still disrupt the gut microbiota (Bäckhed et al., 2015) and may impair immune development (Innis, 2007) and normal metabolism (Chris et al., 2012).

Although the effects on infant gut microbiota composition by feeding methods are well-characterized, to our knowledge, there are few data that compared the differences between mixed-fed (MF), FF, and complementary food-fed (CF) infants with those of BF infants on the composition of the healthy infant gut microbiota and metabolites. Our objective was to show the gut microbiota and metabolites in 77 healthy infants in Northeast China and demonstrate gut microbiome signatures and metabolome characteristic. The observed differences due to feeding methods highlighted the importance of shaping the early gut microbiota and metabolites and may provide theoretical support for the development of infant milk formulations, which are closer to the effects of breast milk on the infant gut microbiota and metabolites.

## Materials and Methods

### Study Design

The study participants were 77 healthy infants from the Northeast region of China. Written informed consent was obtained from the parents or legal guardians of all minor subjects. We used fecal samples to capture transitions from breastfeeding to formula and early introduction of solid food. Mothers were instructed to record the date of the fecal sample and place it in a sterile bag in the refrigerator. To minimize potential confounding effects of early illness or antibiotic administration, we excluded infants who received antibiotic and probiotics. This study was carried out in accordance with the recommendations of the Ethical Committee of Northeast Agricultural University. The protocol was approved by the Ethical Committee of Northeast Agricultural University. All subjects gave written informed consent in accordance with the Declaration of Helsinki. Because of this predetermined exclusion criterion, we also excluded all infants whose mothers have a history of chorioamnionitis and gestational diabetes. Demographic data are provided in Tables S1, S2.

We evaluated infant diet from birth until the time of fecal collection by questionnaires that included questions regarding the duration of breastfeeding, the timing of formula introduction, and the addition of complementary food, if any. Infants who were fed with breast milk and who had never been given formula and complementary food prior to the time of fecal collection were given the status of exclusively BF group. Infants who had been only fed with formula prior to their fecal collection were assigned the status exclusively FF group. Infants who had received both breast milk and formula prior to their fecal collection were identified as MF group. Also, infants who had received complementary food prior to their fecal collection were assigned the status CF group.

### Sample Collection

Fecal samples from diapers of infants were collected and frozen at −20°C immediately upon collection, pending transport, on dry ice, to a −80°C storage facility at Northeast Agricultural University no more than 3 days post collection. Samples were kept at −80°C and thawed once for DNA extraction and metabolomics. A total of 77 fecal samples were collected from 77 infants.

### DNA Extraction and High-Throughput 16S Ribosomal RNA Gene Sequencing

Samples were thawed once, and microbial DNA was extracted using the E.Z.N.A.® Stool DNA Kit (Omega Bio-Tek, Norcross, GA, USA) according to manufacturer's introductions. The V3–V4 hypervariable region of the 16S rRNA gene was amplified by PCR (95°C for 2 min; followed by 27 cycles at 98°C for 10 s, 62°C for 30 s, and 68°C for 30 s; and a final extension at 68°C for 10 min). The first PCR amplified the V3-to-V4 region of the 16S rRNA gene using the 341F and 806R primers 5′-CCTACGGGNGGCWGCAG-3′ (forward primer) and 5′-GGACTACHVGGGTATCTAAT-3′ (reverse primer), where the barcode is an eight-base sequence unique to each sample. PCRs were performed in triplicate 50 μl of mixture containing 5 μl of 10× KOD buffer, 5 μl of 2.5 mM of dNTPs, 1.5 μl of each primer (5 μM), 1 μl of KOD Polymerase, and 100 ng of template DNA.

Amplicons were extracted from 2% agarose gels and purified (Liu et al., 2020) using the AxyPrep DNA Gel Extraction Kit (Axygen Biosciences, Union City, CA, USA) according to the manufacturer's instructions and quantified using QuantiFluor-ST (Promega, USA). Purified amplicons were pooled in equimolar and paired-end sequenced (2 × 250) on an Illumina HiSeq 2500 platform according to the standard protocols.

### Bacterial Community Analysis

Raw data containing adapters or low-quality reads would affect the following assembly and analysis. Thus, to get high-quality clean reads, raw reads were further filtered according to the following rules: (1) removing reads containing more than 10% of unknown nucleotides (N) and (2) removing reads containing <80% of bases with quality (*Q*-value) > 20. Paired-end clean reads were merged as raw tags using FLASH (fast length adjustment of short reads) (Magoč and Salzberg, 2011) (v 1.2.11) with a minimum overlap of 10 bp and mismatch error rates of 2%. Noisy sequences of raw tags were filtered by QIIME (Caporaso et al., 2010) (V1.9.1) pipeline under specific filtering conditions (Bokulich et al., 2013) to obtain the high-quality clean tags.

The effective tags were clustered into operational taxonomic units (OTUs) of ≥97% similarity using UPARSE (Edgar, 2013) pipeline. The tag sequence with highest abundance was selected as representative sequence within each cluster. The abundance statistics of each taxonomic unit were constructed in a Perl script and visualized using SVG software. Alpha diversity was evaluated by richness [abundance-based coverage estimator (ACE) and Chao1] and diversity (Simpson and Shannon). Median estimates are compared across feeding groups using the Kruskal–Wallis test [non-parametric analysis of variance (ANOVA)] and Dunn *post-hoc* tests for multiple comparisons in QIIME (V1.9.1) to identify the complexity of species diversity for each sample. Principal coordinate analysis (PCoA) on unweighted UniFrac distances between the infant microbiota is shown along the first two principal coordinate (PCO) axes. Box-and-whisker plots shown along each PCO axis represent the median and interquartile range with whiskers determined by Tukey's method, indicating the distribution of samples along the given axis. Clustering significance by feeding methods was determined by the Adonis statistical method. OTU rarefaction curve and rank abundance curves were plotted in QIIME (V1.9.1). Statistics of alpha index comparison between groups was calculated by the Welch *t*-test. Alpha index compared among groups was computed by the Kruskal–Wallis *H*-test. The functional group (guild) of the OTUs was inferred using Tax4Fun (Aßhauer et al., 2015) (v1.0).

### Analysis of Metabolites

To investigate differences in the fecal metabolites between different feeding methods, fecal samples from 77 infants were used for further analysis with untargeted metabolomics by liquid chromatography–mass spectrometry (LC-MS). Fifty milligrams of sample was weighted to an Eppendorf (EP) tube. After the addition of 1,000 μl of extract solvent (acetonitrile–methanol–water, 2:2:1, containing internal standard), the samples were vortexed for 30 s, homogenized at 45 Hz for 4 min, and sonicated for 5 min in ice-water bath. The homogenate and sonicate circle were repeated for 3 times, followed by incubation at −20°C for 1 h and centrifugation at 12,000 rpm and 4°C for 15 min. The resulting supernatants were transferred to LC-MS vials and stored at −80°C until the UHPLC-QE Orbitrap/MS analysis. The quality control (QC) sample was prepared by mixing an equal aliquot of the supernatants from all of the samples.

LC-MS/MS analyses were performed using an ultra-high-performance liquid chromatography (UHPLC) system (1290, Agilent Technologies) with a UPLC HSS T3 column (2.1 mm × 100 mm, 1.8 μm) coupled to Q Exactive (Orbitrap MS, Thermo). The mobile phase A was 0.1% formic acid in water for positive and 5 mmol/L of ammonium acetate in water for negative, and the mobile phase B was acetonitrile. The elution gradient was set as follows: 0 min, 1% B; 1 min, 1% B; 8 min, 99% B; 10 min, 99% B; 10.1 min, 1% B; and 12 min, 1% B. The flow rate was 0.5 mL/min. The injection volume was 2 μl. The QE mass spectrometer was used for its ability to acquire MS/MS spectra on an information-dependent acquisition (IDA) basis during an LC/MS experiment. In this method, the acquisition software (Xcalibur 4.0.27, Thermo) continuously evaluates the full scan survey MS data as it collects and triggers the acquisition of MS/MS spectra depending on preselected criteria. Electrospray ionization (ESI) source conditions were set as follows: sheath gas flow rate of 45 Arb, aux gas flow rate of 15 Arb, capillary temperature of 320°C, full MS resolution of 70,000, MS/MS resolution of 17,500, collision energy as 20/40/60 eV in NCE model, and spray voltage of 3.8 (positive) or −3.1 kV (negative), respectively.

MS raw data files were converted to the mzML format using ProteoWizard and processed by R package XCMS (version 3.2). The preprocessing results generated a data matrix that consisted of the retention time (RT), mass-to-charge ratio (m/z) values, and peak intensity. OSI-SMMS (version 1.0, Dalian Chem Data Solution Information Technology Co. Ltd.) was used for peak annotation after data processing with in-house MS/MS database.

For a preliminary visualization of differences among different groups of samples, the unsupervised dimensionality reduction methods principal component analysis (PCA) was applied in all samples using R package models (http://www.r-project.org/). PCA is a statistical procedure that converts hundreds of thousands of correlated metabolites variables into a set of values of linearly uncorrelated variables called principal components.

A variable importance in projection (VIP) score of (orthogonal) partial least squares [(O)PLS] model was applied to rank the metabolites that best distinguished between two groups. The threshold of VIP was set to 1. In addition, *t*-test was also used as a univariate analysis for screening differential metabolites. Those with a *p*-value of *t*-test < 0.05 and VIP ≥ 1 were considered as specific metabolites.

### Pearson Correlation Coefficient Model

The Pearson correlation coefficient between genus level of microbiota and metabolomic datasets were calculated in R (version 3.5.1). The correlation heatmap was generated using pheatmap package in R. The network analysis was performed using igraph package in R.

### Canonical Correspondence Analysis

Canonical correspondence analysis (CCA) was constructed to analyze the influence of selected pathways to microbiota datasets in the genus level, using R vegan package. The influence of metabolome variables on the microbiota community structure was analyzed by the envfit test conducted within the R vegan package.

### Statistics Analysis

All values are performed as the mean ± standard deviation (SD). A minimum of nine independent experiments were carried out for each assay. Analysis of the data was carried out using SPSS 20.0 software (SPSS Inc., Chicago, IL, USA). Statistical differences among groups were determined using one-way ANOVA, followed by Duncan's multiple range test. Values of *p* < 0.05 were considered to be statistically significant.

## Results

### Participant Characteristic

We evaluated the differences in infant feeding methods of 77 appropriately grown infants in Northeastern China by analyzing intestinal microbiome and metabolome. Feeding diaries and telephone surveys were used to assess study participant characteristics. The median gestational age was 38 weeks; all mothers were not given antibiotics and probiotics antepartum and intrapartum (Table 1 and Table S1). Infant age at fecal collection ranged from 16 to 295 days; male infants were 41 of 77 (53.2%); birth weight of all infants was 3,466.8 ± 598.8 g, and the birth height was 50.5 ± 1.3 cm; and there is no significant difference in gestational age, body weight, body height, and infant age. None of the infants had been given antibiotics and probiotics (Table 1 and Table S2).

**Table 1:** Characteristics of the cohort given, stratified by feeding method.

**Characteristics**	**Total (*n* = 77)**	**BF (*n* = 26)**	**MF (*n* = 28)**	**FF (*n* = 14)**	**CF (*n* = 9)**
Infant age, weeks	16.4 ± 9.5	14.1 ± 6.0^a^	12.9 ± 7.7^a^	15.8 ± 6.4^a^	35.1 ± 4.6^b^
Maternal age in years, year	26.9 ± 5.0	27.9 ± 4.6^a^	29.2 ± 4.9^b^	22.4 ± 3.3^b^	24.0 ± 3.1^a^
Gender of baby, *N* (%)
Male	41 (53.2)	20 (76.9)	14 (50.0)	3 (21.4)	4 (44.4)
Female	36 (46.8)	6 (23.1)	14 (50.0)	11 (78.6)	5 (55.6)
Gestational age, weeks	38.7 ± 2.2	38.5 ± 2.4^a^	38.9 ± 1.4^a^	39.0 ± 2.2^a^	38.2 ± 3.9^a^
Mode of delivery, *N* (%)
Vaginal	33 (42.9)	10 (38.5)	13 (46.4)	4 (28.6)	6 (66.7)
Cesarean	44 (57.1)	16 (61.5)	15 (53.6)	10 (71.4)	3 (33.3)
Birth weight, g	3,466.8 ± 598.8	3,509.6 ± 431.3^a^	3,536.6 ± 836.9^a^	3,215.7 ± 429.8^a^	3,516.7 ± 160.1^a^
Birth height, cm	50.5 ± 1.3	50.5 ± 1.3^a^	50.6 ± 1.4^a^	50.0 ± 0.4^a^	50.6 ± 1.3^a^
Parity	1.2 ± 0.4	1.2 ± 0.4^a^	1.1 ± 0.4^a^	1.1 ± 0.1^a^	1.1 ± 0.3^a^
Nationality, *N* (%)
Han	67 (87.0)	25 (96.2)	28 (100.0)	5 (35.7)	9 (100.0)
Manchu	10 (13.0)	1 (3.8)	0 (0.0)	9 (64.3)	0 (0.0)
Antepartum folic acid (%)
Yes	67 (87.0)	24 (92.3)	23 (82.1)	14 (100.0)	6 (66.7)
No	10 (13.0)	2 (7.7)	5 (17.9)	0 (0.0)	3 (33.3)
Maternal education, *N* (%)
College degree or above	33 (42.9)	14 (53.8)	18 (64.3)	1 (7.1)	0 (0.0)
High school and vocational education	21 (27.3)	6 (23.1)	4 (14.3)	9 (64.3)	2 (22.2)
Junior high school education or below	23 (29.9)	6 (23.1)	6 (21.4)	4 (28.6)	7 (77.8)
Work mode, *N* (%)
Full time	36 (46.8)	16 (61.5)	17 (60.7)	2 (14.3)	1 (11.1)
Part-time	41 (53.2)	10 (38.5)	11 (29.3)	12 (84.7)	8 (88.9)

“*±” indicates standard deviation from mean where applicable. Different lowercase letters in the same line indicate significant difference (p < 0.05). Statistical analysis between infants who fed with breast milk (BF), formula (FF), breast milk and formula (MF), or complementary food (CF) was determined using one-way analysis of variance (ANOVA, SPSS 17.0) followed by multiple comparisons with Duncan's multiple range test*.

### Gut Microbiota Compositions Among Various Feeding Methods in Healthy Chinese Infants

**Figure 1:**
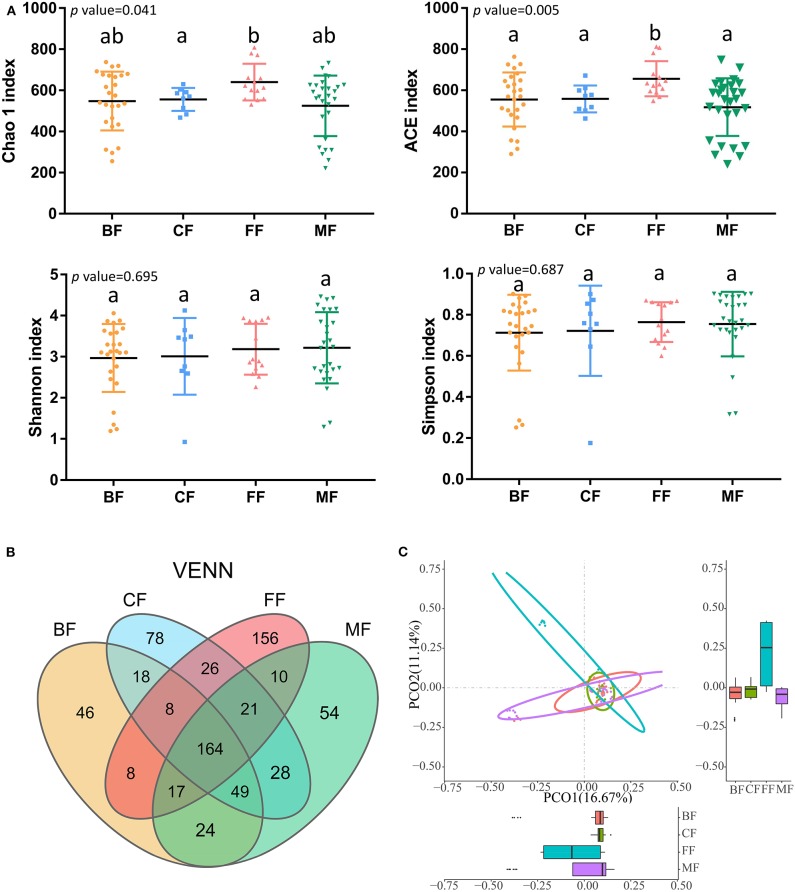
Microbial community structure of infant stool in various feeding methods. **(A)** Alpha diversity in groups. **(B)** Venn diagram of operational taxonomic units (OTUs) among the four groups. **(C)** Principal coordinate analysis (PCoA) on unweighted UniFrac distances in groups; *p* < 0.001, *R*^2^ = 0.1461.

Sequencing yielded a total of 6,762,980 (mean, 87,831; range, 49,892–157,064) bacterial tags, of which 6,417,465 (mean, 83,343; range, 48,366–148,656) passed quality filters (Table S3). The species accumulation curve (Figure S1A) and the rarefaction curve (Figure S1B) of all samples supported the adequacy of the sampling efforts. Following taxonomic assignment, 2,542 OTUs were obtained, and 164 of these OTUs were shared among the four groups. More importantly, we found that the BF, MF, CF, and FF groups all had their unique OTUs, and the amount was 46, 54, 156, and 78, respectively (Figure 1B). This indicated that infants who fed exclusively with breast milk had lower abundance in specific OTUs than had the other infants, and the infants who fed exclusively with formula had the most specific OTUs.

As expected, feeding method was strongly associated with the richness (Chao1 and ACE), diversity (Shannon and Simpson), and composition of gut microbiota (Figure 1A). The richness of microbiota in infants of the FF group was the highest, that of the CF and BF group was lower, and that of the MF group was the lowest. Also, there was significant difference in richness among different feeding methods by comparing Chao1 index (*p* = 0.041) and ACE index (*p* = 0.005); the diversity of microbiota in the BF group was the lowest, higher in the CF and FF groups, and the highest in the MF group; however, there was no significant difference in diversity among the four feeding methods by analyzing the Shannon index (*p* = 0.695) and Simpson index (*p* = 0.687). The community structure of microbiota differed significantly (*p* < 0.01, *R*^2^ = 0.1058), with PCoA on the basis of the unweighted PCoA plots showing clear separation in various groups (Figure 1C). The BF group overlapped almost completely with the MF group, indicating that they exhibited similar microbiota community structures, and was significantly different from the FF (*p* < 0.01, *R*^2^ = 0.1081) and CF groups (*p* < 0.05, *R*^2^ = 0.0521).

### Specific Microbial Phyla and Genera Among Different Feeding Methods

**Figure 2:**
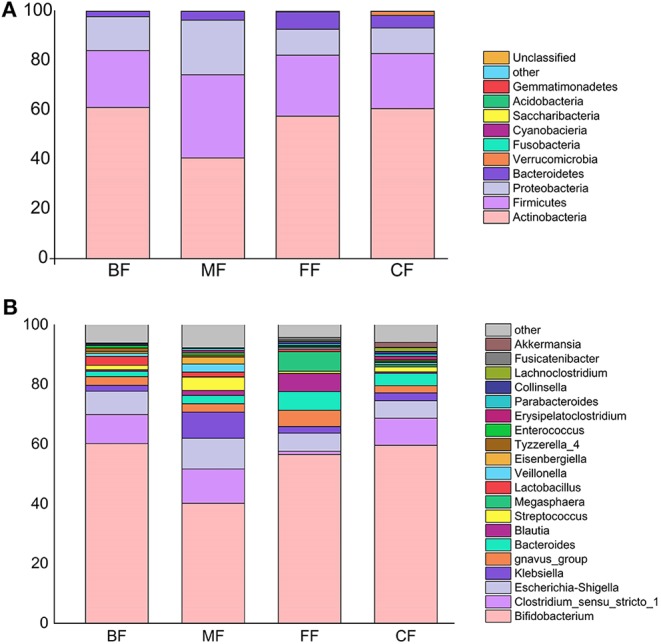
**(A)** Comparison of community composition at the genera level. Only the top 10 most abundant genera are shown for demonstration and clarity. Each column represents a group. **(B)** Comparison of community composition at the phyla level. Only the top 10 most abundant phyla are shown for demonstration and clarity.

Nearly all phyla and genera demonstrated disproportional abundances across different groups, and different feeding methods were observed with particular taxa (Figure 2). Phyla identified in each group were mainly composed of four phyla (more than 98.34% of the sequences), namely, Actinobacteria, Firmicutes, Proteobacteria, and Bacteroidetes. As the most abundant phylum in infant fecal samples of the Northeast China, Actinobacteria's relative abundance was in the order of the BF, CF, FF, and MF groups. In addition, the FF, MF, and CF groups had higher abundance in Bacteroidetes than had the BF group. Firmicutes and Proteobacteria were abundant in the MF group, and Verrucomicrobia was enriched in the CF group (Figure 2A).

A total of 252 bacterial genera were assigned to the four groups. More than 90% of tags were represented by genera including *Bifidobacterium, Clostridium, Escherichia-Shigella, Klebsiella, gnavus_group, Bacteroides, Streptococcus, Blautia, Lactobacillus*, and *Veillonella*, and others representing the remainder (Figure 2B). Although most taxa were similarly abundant among different groups, and the relative abundance of genera was distinct. To further compare the differences in abundance of these genera among the four groups, the Kruskal–Wallis test and Welch *t*-test were performed (Table S4). At the genera level, a total of 20 genera were found to be the most abundant among the four groups, and the significantly different genera were also identified in various groups. The relative abundances of *Bifidobacterium, Lactobacillus, Tyzzerella_4*, and *Enterococcus* in the BF group were higher than those of other groups, but *Klebsiella, Bacteroides, Megasphaera, Erysipelatoclostridium, Parabacteroides, Lachnoclostridium*, and *Akkermansia* were lower. On the other hand, *gnavus_group, Bacteroides, Blautia, Megasphaera, Collinsella*, and *Fusicatenibacter* were enriched in the FF group; *Clostridium_sensu_stricto_1, Escherichia-Shigella, Klebsiella, Streptococcus, Veillonella*, and *Eisenbergiella* were abundant in the MF group, and the CF group was associated with the highest relative abundance of *Lachnoclostridium* and *Akkermansia*.

### Potential Functional Consequences

**Figure 3:**
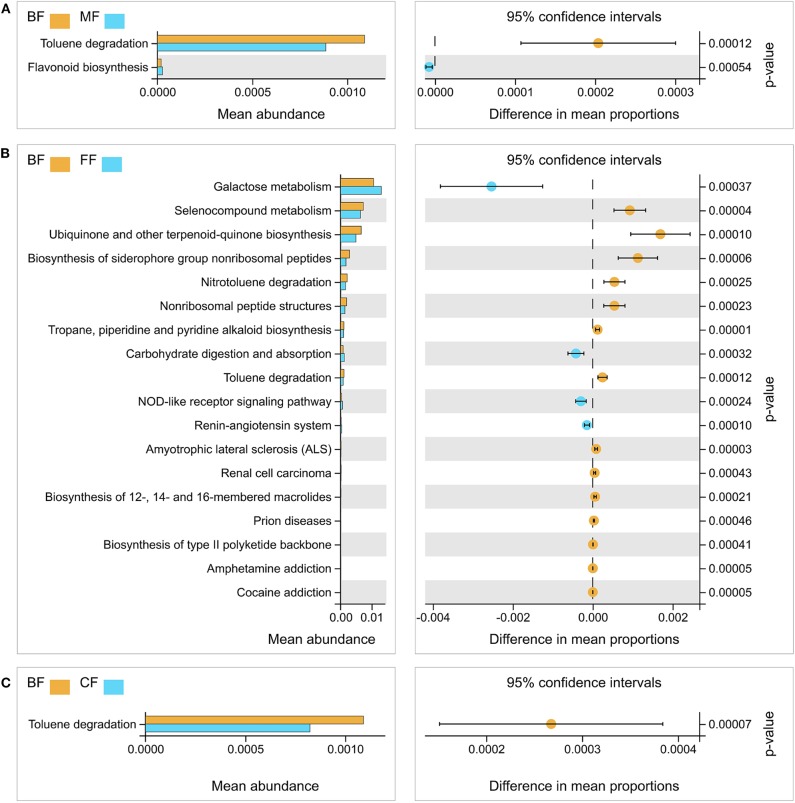
Functional differences analysis between the breastfed (BF) group and the other three groups, respectively. **(A)** The BF group vs. the mixed-fed (MF) group. **(B)** The BF group vs. the formula-fed (FF) group. **(C)** The BF group vs. the CF group.

PCoA analysis was conducted to analyze the functional content similarity in all samples (i.e., data explained 85.7% of the variation), and the results demonstrated that different feeding methods had different functions of bacterial community (Figure S2). Furthermore, the functional profiles of gut microbial communities from all samples were predicted using Tax4Fun and analyzed by the Welch *t*-test. The results demonstrated that most of the predicted pathways of the MF, FF, and CF groups were still significantly different from those of the BF group, especially the toluene degradation, nitrotoluene degradation, ubiquinone, other terpenoid-quinone biosynthesis, and selenocompound metabolism (Figure 3 and Table S5).

### Global Overview of Gut Metabolism in the Fecal Metabolome of the Various Feeding Methods

**Figure 4:**
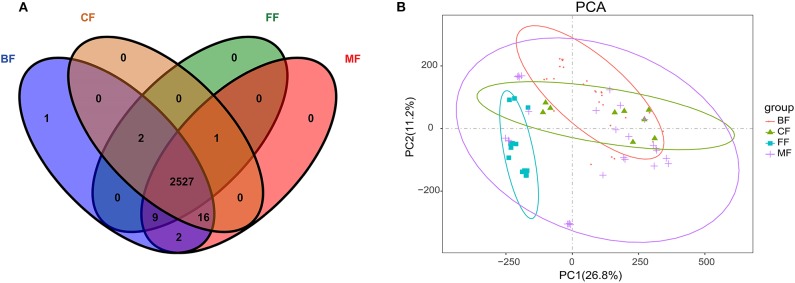
**(A)** Venn diagram of metabolites among all groups. **(B)** Principal component analysis (PCA) diagram of metabolites in all fecal samples.

Owing to the specific microbiota associated with feeding methods, we hypothesized that alterations in metabolic pathways may be at least partially affected by gut microbiota in different feeding methods. Therefore, we subsequently performed metabolome analysis of fecal samples using untargeted metabolomics approach. Metabolites were assessed by positive ion mode (POS) and negative ion mode (NEG) in LC-MS detection to reveal the effects of metabolites by feeding methods. We successfully identified 2,558 different metabolites under the POS and NEG ion mode in which 2,527 were shared (Figure 4A and Table S6). We also found one metabolite that was detectable in the BF group; and there was one metabolite, three metabolites, 19 metabolites, and 12 metabolites that were not detectable in the BF, MF, FF, and CF groups, respectively (Figure 4A). PCA clearly showed differences between the BF, MF, FF, and CF groups on the basis of the first two principal components, PC1 (26.8%) and PC2 (11.2%), suggesting differences in metabolites abundance and signatures by the various feeding methods (Figure 4B).

All metabolites were mapped onto 106 different Kyoto Encyclopedia of Genes and Genomes (KEGG) metabolic pathways including carbohydrate metabolism (265 metabolites), lipid metabolism (96 metabolites), amino acid metabolism (178 metabolites), metabolism of other amino acids (82 metabolites), nucleotide metabolism (235 metabolites), metabolism of cofactors and vitamins (173 metabolites), and energy metabolism (80 metabolites) (Figure S3).

### Clustering and Correlation Reveal Discriminatory Metabolite Among the Four Groups

**Figure 5:**
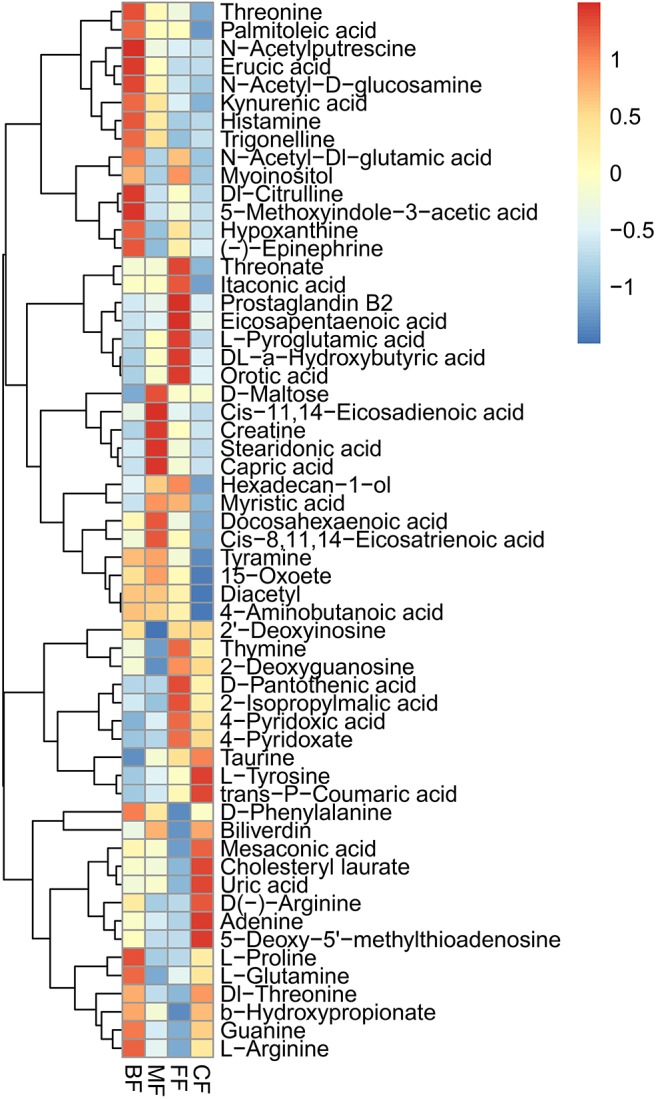
Heatmap of the most abundant metabolites of four feeding methods, as identified by variable importance in projection (VIP) scores in partial least squares discriminant analysis (PLS-DA). Every column represents a different metabolite in stool samples. Higher abundances are marked in red color, whereas lower abundances in blue color.

There were 146 specific metabolites affected by feeding method (Table S7). Furthermore, we analyzed the specific metabolites with metabolic pathways, and a heatmap was used to visualize the results of these metabolites (Figure 5), such as the BF group, which showed higher abundance of threonine, *N*-acetylputrescine, *N*-acetyl-d-glucosamine, kynurenic acid, histamine, trigonelline, *N*-acetyl-dl-glutamic acid, dl-citrulline, 5-methoxyindole-3-acetic acid, hypoxanthine, d-phenylalanine, l-proline, l-glutamine, guanine, and l-arginine; d-maltose, *cis*-11,14-eicosadienoic acid, creatine, stearidonic acid, capric acid, myristic acid, docosahexaenoic acid, *cis*-8,11,14-eicosatrienoic acid, tyramine, and 15-oxoete were demonstrated to be more enriched in the MF group; the FF group showed higher abundance of myoinositol, threonate, itaconic acid, prostaglandin B2, eicosapentaenoic acid, l-pyroglutamic acid, dl-α-hydroxybutyric acid, orotic acid, hexadecane-1-ol, thymine, 2-deoxyguanosine, d-pantothenic acid, 2-isopropylmalic acid, 4-pyridoxic acid, and 4-pyridoxate; taurine, l-tyrosine, trans-*p*-coumaric acid, mesaconic acid, cholesteryl laurate, uric acid, d(–)-arginine, adenine, 5-deoxy-5′-methylthioadenosine, and dl-threonine were validated to be more abundant in the CF group. More importantly, we found the metabolite named pachymic acid was uniquely present in the BF groups, and other groups did not have unique metabolites. Taken together, our data clearly and robustly showed that different feeding methods presented specific metabolites.

**Figure 6:**
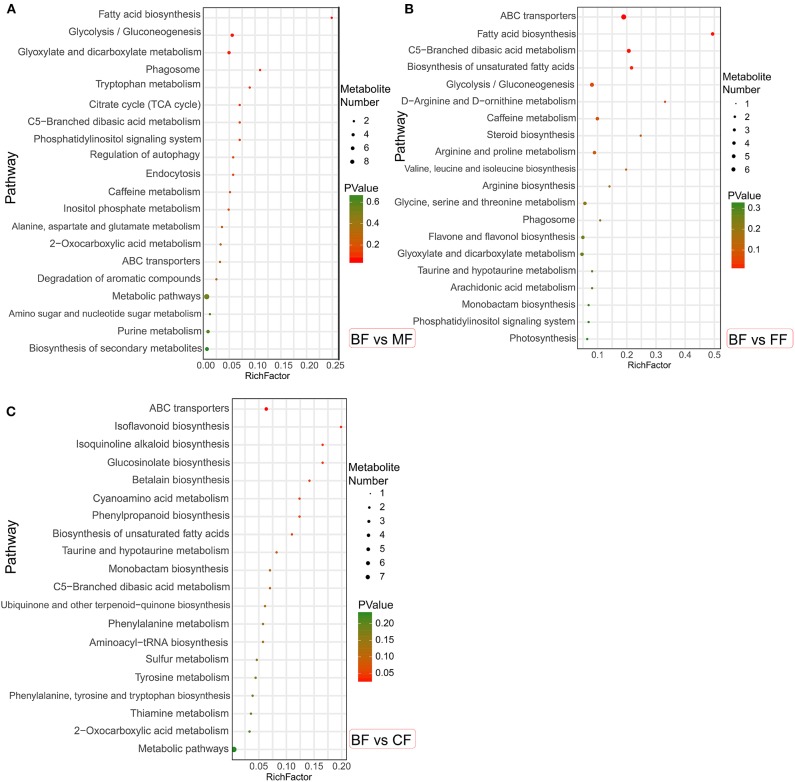
The bubble diagrams of metabolic pathways in level 3. **(A)** The mixed-fed (MF) group vs. the breastfed (BF) group. **(B)** The formula-fed (FF) group vs. the BF group. **(C)** The complementary food-fed (CF) group vs. the BF group.

These specific metabolites were mapped onto different KEGG metabolic pathways (Figure 6). The MF, FF, and CF groups were different from the BF group: in particular, compared with the BF group, the MF group was more significantly enriched in fatty acid biosynthesis; the FF group was significantly abundant in ABC transporters, fatty acid biosynthesis, C5-branched dibasic acid metabolism, and biosynthesis of unsaturated fatty acids; and the CF group was significantly improved in ABC transporters, isoquinoline alkaloid biosynthesis, and glucosinolate biosynthesis. It was revealed that other groups were still different from the BF group, especially with regard to the fatty acid biosynthesis and ABC transporters.

### The Associations Between Infant Feeding, Gut Microbiota, and Metabolites

**Figure 7:**
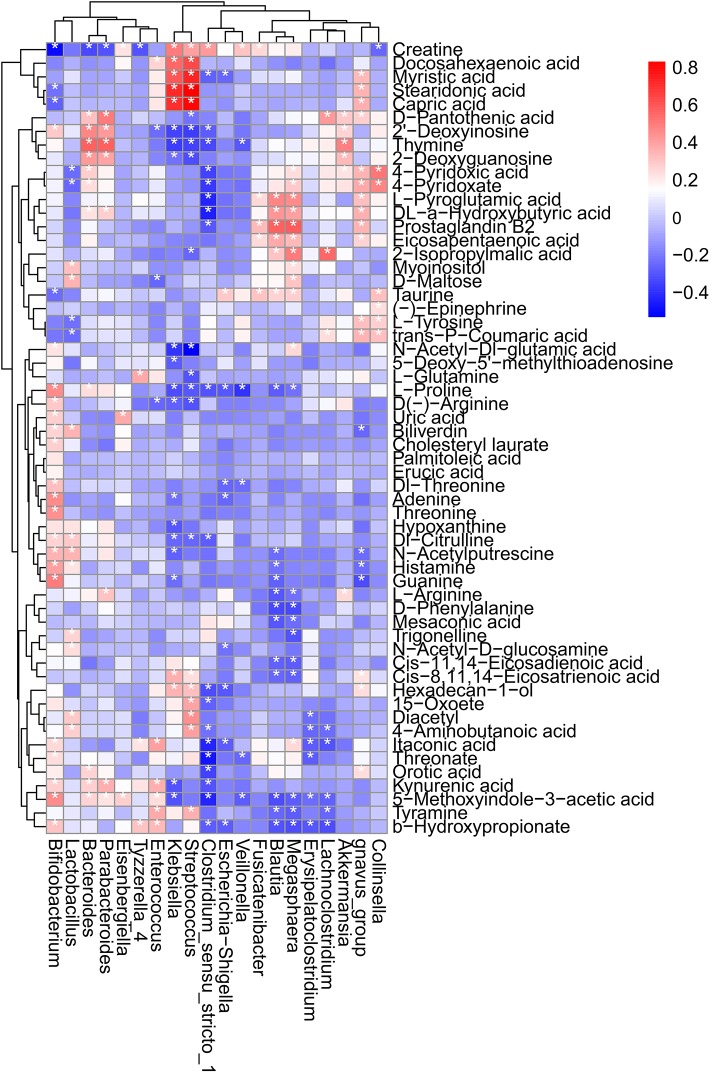
Heatmap diagram representing the correlation between the top 20 microbiota and specific metabolites of the four feeding methods. *p < 0.05.

We analyzed which dominant microbiota caused the differences in metabolites. We also analyzed the association for the top 20 microbiota and specific metabolites (Figure 7). Further analysis showed that *Bifidobacterium* was significantly positively related to l-proline, d(–)-arginine, dl-threonine, adenine, threonine, dl-citrulline, *N*-acetylputrescine, histamine, guanine, and threonate while significantly negatively related to creatine, capric acid, and taurine. *Lactobacillus* was significantly positive correlated with d-maltose, dl-citrulline, *N*-acetylputrescine, and histamine while significantly negatively correlated with l-tyrosine and trans-*p*-coumaric acid. *Bacteroides* was positively associated with thymine, l-proline, and orotic acid while negatively associated with creatine. Also, capric acid, myristic acid, docosahexaenoic acid, and creatine were more positively correlated with *Klebsiella*; however, guanine, *N*-acetylputrescine, dl-citrulline, adenine, d(–)-arginine, l-proline, and thymine were more negatively associated with *Klebsiella*.

**Figure 8:**
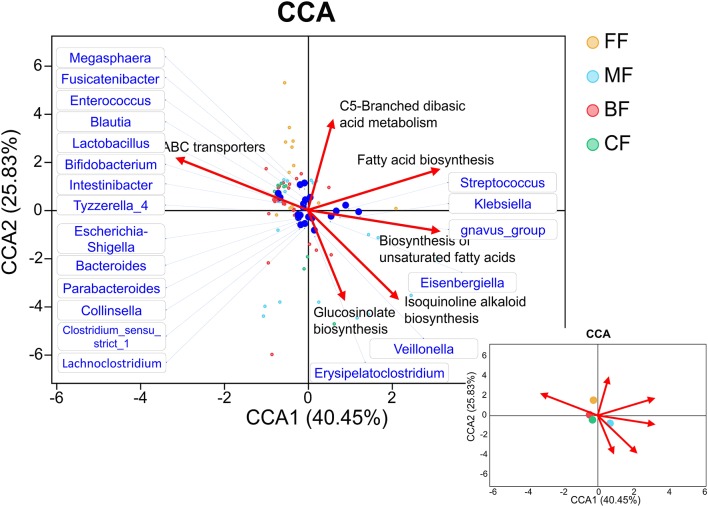
Canonical correspondence analysis (CCA) diagram that showed the associations between infant feeding, gut microbiota, and metabolites. The blue dots represent the gut microbiota correlated with the pathways; the lower right picture shows the relationship between pathways and groups.

Subtle but statistically significant differences in the microbiota and metabolites were observed in four feeding methods. We analyzed the correlation between gut microbiota and the specific KEGG metabolic pathways, which were significantly enriched in other groups compared with the BF group. These KEGG metabolic pathways have dissimilar effects on the distribution of species (Figure 8). Apparently, the BF group was positively correlated with ABC transporters, and the CF group was more similar with the BF group. However, the FF group was positively related to ABC transporters and C5-branched dibasic acid metabolism and was negatively related to fatty acid biosynthesis and biosynthesis of unsaturated fatty acid. On the contrary, the MF group was positively correlated with fatty acid biosynthesis and biosynthesis of unsaturated fatty acid, whereas it was negatively correlated with ABC transporters. ABC transporters were positively related to *Bifidobacterium, Lactobacillus, Bacteroides*, and *Parabacteroides* and was negatively related to *Klebsiella*. On the contrary, fatty acid biosynthesis and biosynthesis of unsaturated fatty acid were more positively associated with *Klebsiella* and were more negatively associated with other bacteria.

### The Comparison of Functional Differences Between Metabolites Enriched and Microbiota Predicted

**Figure 9:**
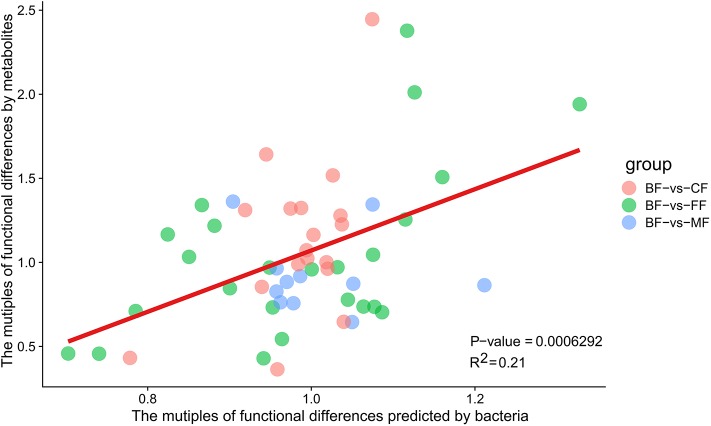
The liner regression diagram of predicted functional differences multiples by bacteria and functional differences multiples of metabolites.

The functional differences multiples of microbiota predicted by Tax4Fun and the functional differences multiples enriched by metabolites were closely distributed over both sides of the line (Figure 9 and Table S8). It was revealed that between the BF group and the other three groups, the functional differences predicted were more similar to the functional differences enriched by metabolites. It was revealed that microbiota was strictly related to the metabolites, and the distinction of microbiota caused the difference in the metabolites.

## Discussion

In this study, we validated the associations for gut microbiota and metabolites by feeding methods from 77 healthy Chinese infants. Although the effects of breast milk and formula on the development of infant gut microbiota are well-known (Paolella and Vajro, 2016; Milani et al., 2017; Pannaraj et al., 2017; Timmerman et al., 2017), to our knowledge, our findings have been rarely explored in previous studies and identified differences according to the feeding methods, especially the distinction among the formula feeding, the mixed feeding, and the complementary food feeding with the exclusive breastfeeding in gut microbiota and metabolites. We also revealed the gut microbiota related to the differential metabolites. Furthermore, we compared the functions of gut microbiota and metabolites. We found that the abundance of main genera was strongly distinct and that some of the metabolites in various feeding methods were different significantly.

Our results demonstrated that the bacterial species richness was higher in the order of the FF, CF, BF, and MF groups. Moreover, the bacterial species diversity was higher in the order of the MF, FF, CF, and BF groups. These findings are in conformity with a recent report that decreased bacterial species richness or diversity in exclusively BF vs. FF (Wood et al., 2018) and are inconsistent with the report that richness and diversity of the MF infants are between the BF and the FF infants (Forbes et al., 2018). Microbial community structures of various feeding methods also differed significantly, which was the same with the previous report (Thompson et al., 2015).

The main health-relevant genera were *Bifidobacterium* and *Lactobacillus* in all groups. In our study, the relative abundance of *Bifidobacterium* and *Lactobacillus* species was more abundant in the BF group compared with other groups. Also, the MF infants had a lower relative abundance of *Bifidobacterium* than the FF infants. It was different from the previous reports (Madan et al., 2016). The relative abundance of *Lactobacillus* was in accordance with the recent reports that it was higher in the BF infants than in the MF infants and that it was also higher in the MF infants than the FF infants (Madan et al., 2016). Moreover, the CF infants had the lowest relative abundance of *Lactobacillus* than had the other groups. *Bifidobacterium* is present naturally in the gastrointestinal tract of healthy human, making them a common marker for prebiotic capacity. There have been numerous reported studies and reviews on the increase in *Bifidobacterium*, which is considered beneficial because of its association with many positive health outcomes (Amarri et al., 2006; Carlson and Slavin, 2016; Laursen et al., 2016; Paolella and Vajro, 2016; Pannaraj et al., 2017; Qasem et al., 2017; Timmerman et al., 2017). Like *Lactobacillus*, these bacteria are saccharolytic, often considered a beneficial trait (Salyers, 1979). *Bifidobacterium* and *Lactobacillus* have been reported to improve diarrheal outcomes in infants (Saavedra et al., 1994; Guandalini et al., 2000; Chouraqui et al., 2004; Corrêa et al., 2005; Zvi et al., 2005; Roberto Berni et al., 2007). Increases in *Lactobacillus* are also considered as a beneficial effect. Human milk provides optimal infant nutrition (Schwarzenberg et al., 2018) and favors *Bifidobacterium* and *Lactobacillus* spp. (Bäckhed et al., 2015; Planer et al., 2016). However, many of these studies did not compare the partial breastfeeding mixed with formula and introduction of complementary foods with breast milk and formula. The result showed that the infants who were fed with breast milk, compared with other feeding methods, were more enriched in *Bifidobacterium* and *Lactobacillus*.

Our findings elucidated that the relative abundance of *Bacteroides* in BF group was the lowest and higher in the MF and CF groups, whereas it was highest in the FF group. The relative abundance of *Bacteroidetes* in gut microbiota is highly susceptible to dietary changes (Gorvitovskaia et al., 2016). *Bacteroidetes* can be transmitted during maternal delivery; hence, they are the original members of gut microbiota during lactation (Reid, 2004). They are related to animal protein and saturated fat diet and also have specific carbohydrate enzymes. However, some strains of the genus are virulent pathogens, such as *Bacteroides thetaiotaomicron* and *Bacteroides distasonis*, which can cause diseases such as inflammatory enteritis diarrhea or bacteremia (Boquet et al., 1998). Our findings also showed that the relative abundance of *Klebsiella* in the BF group was the lowest and higher in the FF and CF groups, whereas it was the highest in the MF group. *Klebsiella* is a gram-negative bacterium; in particular, *Klebsiella pneumoniae* is an important opportunistic pathogen and one of the iatrogenic infectious bacteria. The high relative abundance of *Klebsiella* was more often related to infection (Wongsurakiat and Chitwarakorn, 2019). They are mainly *K. pneumoniae, Klebsiella ozaenae*, and *Klebsiella cleromatis*. Differences in gut microbiota composition of feeding methods were also evident from a lot of studies. Such differences in microbiota composition were related to the differences in the gut metabolites.

Our observations showed that dl-citrulline, *N*-acetylputrescine, histamine, threonine, l-proline, l-glutamine, guanine, and l-arginine were more enriched in the BF group; that taurine, l-tyrosine, d(–)-arginine, adenine, and dl-threonine were validated to be more abundant in the CF group; and that d-maltose, creatine, stearidonic acid, capric acid, myristic acid, and docosahexaenoic acid were demonstrated to be more enriched in the MF group. Also, the FF group have higher abundance of itaconic acid, eicosapentaenoic acid, l-pyroglutamic acid, orotic acid, and thymine. It is well-accepted that the metabolite profile of the gut lumen is dependent on the structure and function of the resident microbiota (Nauta et al., 2013; Donia and Fischbach, 2015). Our findings showed that *Bifidobacterium* was significantly positive related to l-proline, d(–)-arginine, dl-threonine, adenine, threonine, dl-citrulline, *N*-acetylputrescine, histamine, guanine, and threonate, whereas it was significantly negatively related to creatine, capric acid, and taurine. Proline increased the relative abundance of *Bifidobacterium* (Ji et al., 2018). Kitada showed that the level of putrescine, a polyamine found abundantly in the human intestinal lumen, is increased in the colonic lumen following administration of arginine and the probiotic *Bifidobacterium* (Kitada et al., 2018). *Lactobacillus* was significantly positive correlated with d-maltose, dl-citrulline, *N*-acetylputrescine, and histamine, whereas it was significantly negative correlated with l-tyrosine and trans-*p*-coumaric acid. *Bacteroides* was positively associated with thymine, l-proline, and orotic acid, whereas it was negatively associated with creatine. Also, capric acid, myristic acid, docosahexaenoic acid, and creatine were more positively correlated with *Klebsiella*; however, guanine, *N*-acetylputrescine, dl-citrulline, adenine, d(–)-arginine, l-proline, and thymine were more negatively associated with *Klebsiella*. Ji reported that dietary supplementation with 1% proline decreased the amounts of *K. pneumoniae* (Ji et al., 2018). The amino acid metabolic pathway was more related to infants who fed exclusively with breast milk. This was consistent with the previous report showing enriched arginine in mother-fed relative to FF piglets (Poroyko et al., 2010) and was the same with the report in which amino acid synthesis pathways were increased in the microbiota of BF infants (Baumanndudenhoeffer et al., 2018). Compared with the BF group, the MF and FF groups were more related to fatty acid biosynthesis and biosynthesis of unsaturated fatty acid. It was opposite with the result of Nhan (Ho et al., 2018). These findings may provide insight into biological mechanisms for the adverse health outcomes of children that were not BF or non-exclusively BF in early months of life (Stuebe, 2009; Cardwell et al., 2012;Yan et al., 2014).

## Conclusions

Overall, understanding the patterns of microbial colonization and metabolite composition of healthy infants is critical for determining the health effects of specific alterable early-life risk factors and exposures. Our findings compared the distinction between FF, MF, CF, and BF infants. Moreover, our study identified the bacteria that induced the metabolites changes, suggesting that differences of feeding methods in gut microbiota influence the metabolite milieu. The differences between exclusively BF infants and other infants were the abundance of *Bifidobacterium* and *Lactobacillus* and the abundance of dl-citrulline, threonine, l-proline, l-glutamine, guanine, and l-arginine. The exclusively BF infants had the highest abundance of *Bifidobacterium, Lactobacillus*, dl-citrulline, threonine, l-proline, l-glutamine, guanine, and l-arginine than had the MF infants, the exclusively FF infants, and the CF infants. Furthermore, compared with the exclusively BF infants, the MF infants and the exclusively FF infants were more abundant in fatty acid biosynthesis. These results open avenues to explore the gut microbiome–metabolome associations for biomarker discovery and to identify important areas for future research.

## Data Availability Statement

The raw sequence datasets from 16S ribosomal RNA gene sequencing are available in the SRA database under accession number PRJNA562650.

## Ethics Statement

The studies involving human participants were carried out in accordance with the recommendations of the Ethical Committee of Northeast Agricultural University. Written informed consent to participate in this study was provided by the participants' legal guardian/next of kin.

## Author Contributions

NL, BL, and GH conceived the study and designed the project. NL, FY, and GH helped to collect fecal samples of infants and investigate infant information. NL, YS, YY, and JG performed the experiment. NL, BL, and NW analyzed the data and drafted the manuscript. BL helped to revise the manuscript. All authors have read and agreed to the published version of the manuscript.

## Conflict of Interest

The authors declare that the research was conducted in the absence of any commercial or financial relationships that could be construed as a potential conflict of interest.
