# Supplementary material for: Distinct Gut Microbiota and Metabolite Profiles Induced by Different Feeding Methods in Healthy Chinese Infants
Source: Front Microbiol. 2020 May 6;11:714. doi: 10.3389/fmicb.2020.00714 (PMC7219020; doi:10.3389/fmicb.2020.00714)
Supplement: Supplementary file 1 [file Data_Sheet_1.pdf]

Supplementary Material

1. Supplementary Tables

Table S1 Basic information of the mothers.

Table S2 Basic information of the infants.

Table S3 Bacterial libraries after taxonomic assignment of OTUs at the 97 % minimum similarity level.

Table S4 Comparison of specific species in various feeding methods.

Table S5 Differences of functions compared between the MF, FF and CF groups with the BF group.

Table S6 Metabolites that assessed by positive ion mode (POS) and negative ion mode (NEG) in LC-MS detection.

Table S7 Metabolites that affect by feeding methods and were significantly different in various feeding methods.

Table S8 The comparison of functional differences between metabolites enriched and microbiota predicted.

2. Supplementary Figures

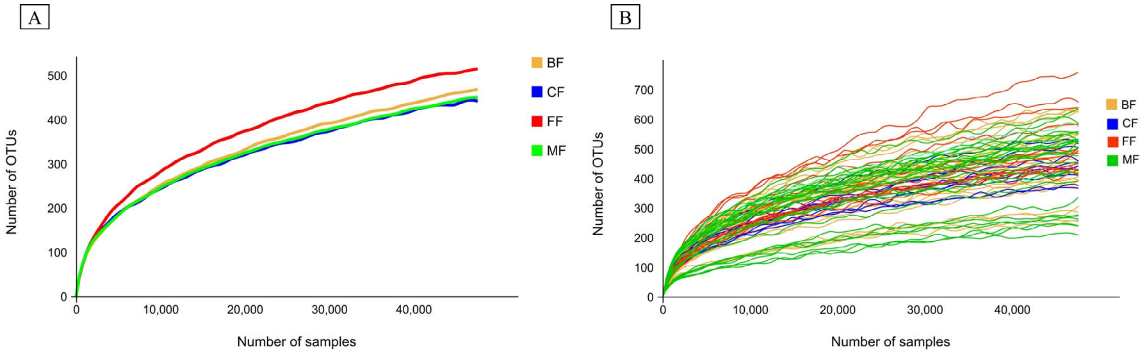

Figure S1. The species accumulation curve and the rarefaction curve of all samples.

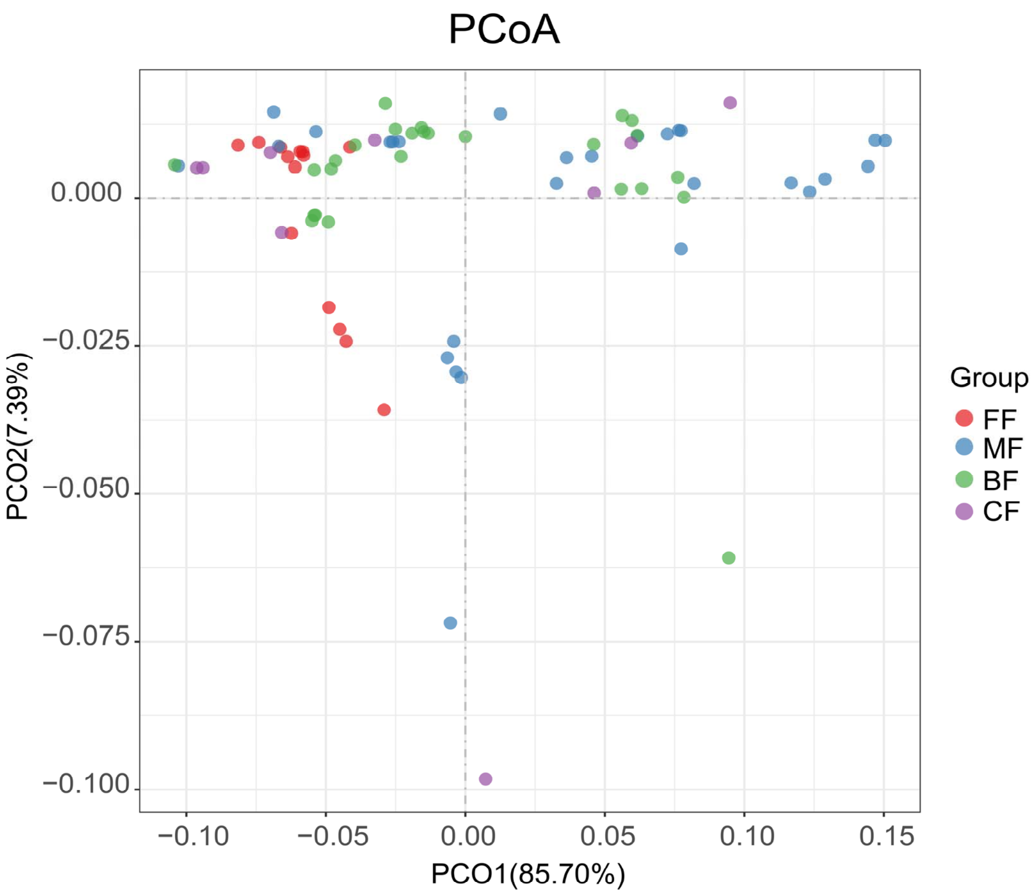

Figure S2. The diagram of PCoA analysis in functions of all samples.

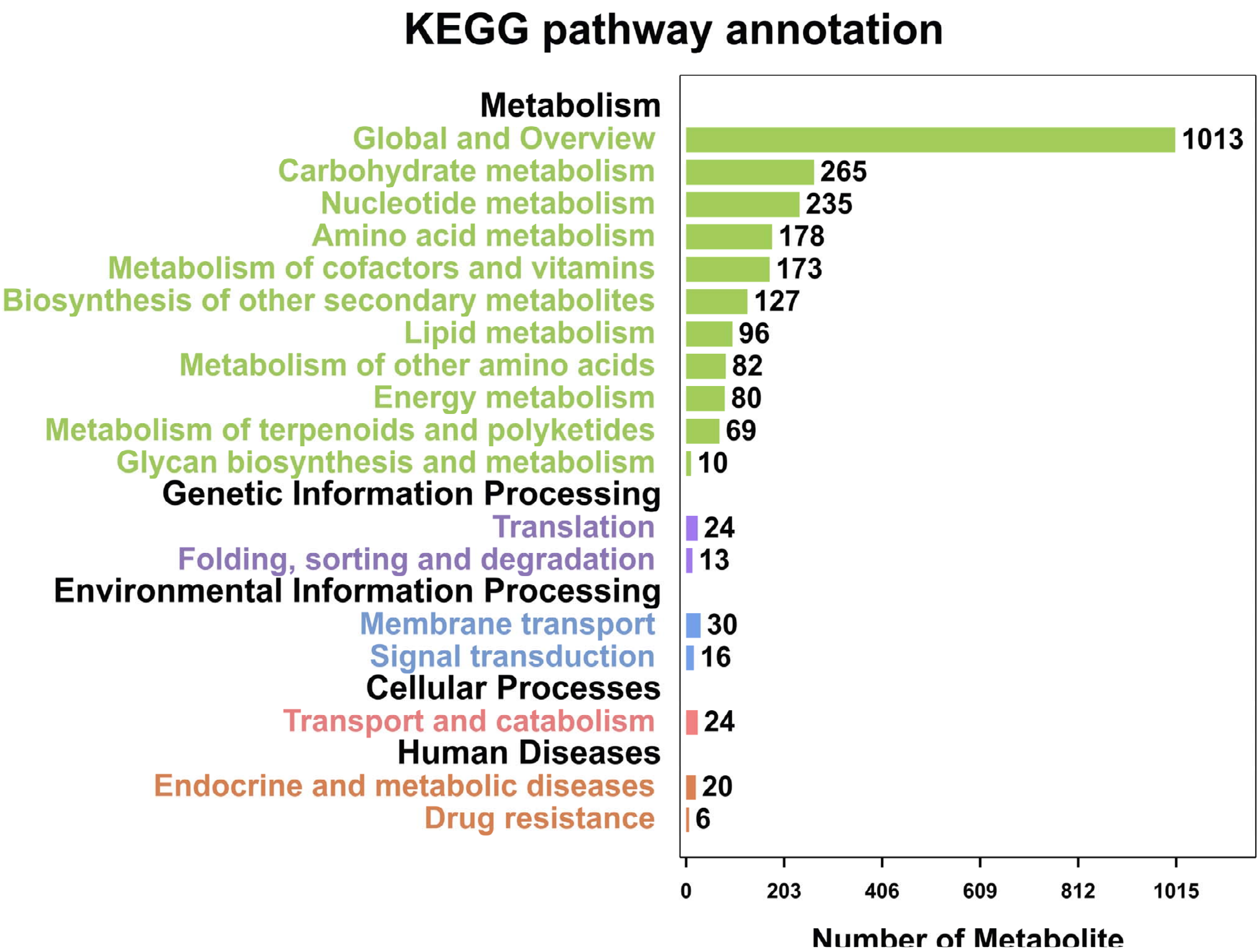

Figure S3. The diagrams of metabolic pathways of all samples in Level 2.
